# Artificial Neural Network to Predict Vine Water Status Spatial Variability Using Multispectral Information Obtained from an Unmanned Aerial Vehicle (UAV)

**DOI:** 10.3390/s17112488

**Published:** 2017-10-30

**Authors:** Tomas Poblete, Samuel Ortega-Farías, Miguel Angel Moreno, Matthew Bardeen

**Affiliations:** 1 Centro de Investigación y Transferencia en Riego y Agroclimatología (CITRA), Universidad de Talca, Casilla 747, Talca 3460000, Chile; totopoblete@gmail.com; 2 Research program on Adaptation of Agriculture to Climate Change (A2C2), Universidad de Talca, Casilla 747, Talca 3460000, Chile; mbardeen@utalca.cl; 3 Regional Centre of Water Research, University of Castilla-La Mancha, Campus Universitario s/n, 02071 Albacete, Spain; miguelangel.moreno@uclm.es; 4 Facultad de Ingeniería, Universidad de Talca, Curicó 3340000, Chile

**Keywords:** multispectral image processing, artificial neural network, UAV, midday stem water potential

## Abstract

Water stress, which affects yield and wine quality, is often evaluated using the midday stem water potential (Ψ_stem_). However, this measurement is acquired on a per plant basis and does not account for the assessment of vine water status spatial variability. The use of multispectral cameras mounted on unmanned aerial vehicle (UAV) is capable to capture the variability of vine water stress in a whole field scenario. It has been reported that conventional multispectral indices (CMI) that use information between 500–800 nm, do not accurately predict plant water status since they are not sensitive to water content. The objective of this study was to develop artificial neural network (ANN) models derived from multispectral images to predict the Ψ_stem_ spatial variability of a drip-irrigated Carménère vineyard in Talca, Maule Region, Chile. The coefficient of determination (R^2^) obtained between ANN outputs and ground-truth measurements of Ψ_stem_ were between 0.56–0.87, with the best performance observed for the model that included the bands 550, 570, 670, 700 and 800 nm. Validation analysis indicated that the ANN model could estimate Ψ_stem_ with a mean absolute error (MAE) of 0.1 MPa, root mean square error (RMSE) of 0.12 MPa, and relative error (RE) of −9.1%. For the validation of the CMI, the MAE, RMSE and RE values were between 0.26–0.27 MPa, 0.32–0.34 MPa and −24.2–25.6%, respectively.

## 1. Introduction

The largest wine producing and growing regions (France, Spain, Australia, South Africa, parts of USA, Chile and Argentina) have experienced water scarcity during the last years [1,2]. Under this scenario, modern irrigation management is required to improve water productivity (wine production per unit of applied water, kg·m^−3^) of viticultural areas. Traditionally, irrigation management has been based on the monitoring of vine evapotranspiration, soil water content and physiological plant responses. Although some of these methods have been widely used, they do not consider adequately the spatial variability of soil, cultivar and climate conditions to schedule irrigation. In this regard, site-specific irrigation management (SSIM) can be used as a tool to improve water productivity [3,4]. In that case, SSIM characterizes the effect of the intra-vineyard spatial variability of soil and canopy vigor on the estimation of irrigation scheduling (irrigation frequency and timing).

### 1.1. Monitoring of Evapotranspiration, Soil Water Content and Physiological Plant Responses

Usually, irrigation management relies on the estimation of actual evapotranspiration (ETa) [1]:

Eta = Kc × ETo(1)

where ETo is the reference evapotranspiration (mm·day^−1^) and Kc is the crop coefficient. ETo is calculated using the Penman–Montieth model which requires air temperature, relative humidity, wind speed, and solar radiation as inputs [1,2]. The Kc is the ratio between ETa and ETo, where ETa can be measured using a lysimeter, soil water balance approach, eddy covariance method, Bowen ratio energy balance system, or surface renewal method [5]. Due to the specificity of Kc for local conditions, it is necessary to calibrate this parameter to the specific conditions of vineyards [6]. To solve this problem, Turner [7] has suggested monitoring the soil water content to schedule irrigation.

Some of the disadvantages of using soil water content monitoring in vineyards are: (1) it does not represent water consumption in vines with extensive root systems and when vines are grown in deep soil; and (2) it does not predict water content in soil with high salinity and a high percentage of stone [8]. In this regard, Granier, et al. [9] indicated that measurement of physiological parameters provides better information about the whole plant, controlling systems to climatic conditions and atmospheric water demands compared with soil water content.

Among the most studied technologies for monitoring physiological plant responses to water stress are sap flow measurement [10,11], dendrometry [12], gas exchange [13,14], chlorophyll (Chl) fluorescence [15], stomatal conductance [15], canopy temperature [16,17,18,19] and plant water potential [20,21]. The measurement of plant water potential is a good predictor of vine water status and has been the most commonly used technique to characterize water stress for vineyards under regulated deficit irrigation (RDI) conditions [22,23,24]. The midday stem water potential (Ψ_stem_) has been proposed as the most reliable technique to schedule the irrigation of orchards and vineyards [25,26]. However, the measurement of Ψ_stem_ must be carried out in the field for each plant, which incurs high costs and does not capture the spatial variability of water status [27,28,29]. Based on that, remote sensing platforms have been proposed to replace ground-based measurements and to assess the spatial variability of water status in larger areas [28,30].

### 1.2. Remote Sensing and Multispectral Indices to Assess Spatial Variability

Different types of remote sensing platforms can be used to carry different types of sensors to assess different spectral wavelength ranges [31]. Unlike manned vehicles and satellite, unmanned aerial vehicles (UAV) have several advantages: they are inexpensive, flexible, more independent of climatic variables and can be flown with minimal training [32]. All these characteristics allow us to obtain high resolution information in an automatic and accurate manner. By using these vehicles it is possible to transport sensors, which provide multispectral information that can be integrated into spectral indices to predict several physiological variables [28,33,34,35], specifically for the evaluation of intra-vineyard spatial variability [35,36,37,38]. Some researchers have suggested that several indices using information between 500 and 800 nm can estimate vine water status indirectly, with coefficients of determination (R^2^) ranging between 0.01 and 0.68 (Table 1).

For example, values of R^2^ for the TCARI/OSAVI ranged from 0.58 in Tempranillo [28] to 0.01 in Thompson Seedless [38], while those for PRI varied from 0.53 in Thompson Seedless [38] to 0.19 in Cabernet Sauvignon [37]. For the NDVI, Baluja, et al. [28] indicated R^2^ = 0.68 in Tempranillo while Rapaport, et al. [37] observed R^2^ = 0.03 in Cabernet Sauvignon.

In this regard, some studies suggest that spectral indices based on information between 500 and 800 nm are not suitable in all field conditions to estimate vine water potential and suggest that wavelengths greater than 800 nm could better represent water status [37,39]. For example, WI (water Index = R900/R970) has presented good correlations with water status variables in chardonnay with R^2^ values of 0.81 and 0.95 in non-stressed and stressed vines, respectively [36]. Several researchers have suggested that hyperspectral information can be adapted to predict water status and physiological parameters. In that context, Rapaport, et al. [37] indicated that WABIs (water balance indices) that use visible (VIS) and short waver infrared (SWIR) information are good predictors to identify water stress in grapevines showing the best correlation (R^2^ = 0.89) for the WABI-2 index (R1500 – R538)/(R1500 + R538). Rallo, et al. [40] suggested that information between SWIR and NIR improves the prediction of leaf water potential over the visible spectrum with a R^2^ of 0.7 in the validation process. Also, Pôças, et al., in [41,42] showed that developing different modelling techniques using wavelength information of VIS, green, red-edge and NIR can predict water status with good correlations (R^2^ = 0.79 and R^2^ = 0.78–0.80, respectively). Rodríguez-Pérez, et al. [43] predicted water status using hyperspectral information with R^2^ = 0.91 for EWT (equivalent water thickness) and R^2^ = 0.619 for water potential. Based on these relationships between spectral information and water status, the use of miniaturized hyperspectral and multispectral sensors has been proposed to be mounted on an UAV [44]. Hyperspectral sensors can provide a measure of spectral response across hundreds of narrowly defined spectral bands simultaneously, however, they sacrifice spatial resolution and their commercial prices remain high [45]. Hyperspectral sensors also possess inherent image distortion causing geometric errors and limit detection of vegetation stress using red-edge information [5]. On the other hand, multispectral cameras, specifically the MCA-6 camera (Tetracam’s miniature camera array), can detect a VIS-NIR range of the electromagnetic spectrum and has been proven in several studies to identify different types of stress in plants [28,33,46,47,48,49]. Also, the spectral reflectance of this type of camera has been compared with WorldView-2 satellite, producing similar results [50].

### 1.3. Machine Learning Techniques and ANN

As conventional multispectral indices present limitations to assess water, artificial neural networks (ANN) could be used to improve the assessment of the spatial variability of vine water status spatial variability. Machine learning techniques and ANN models are applied to perform regression analyses of highly nonlinear problems and find nonlinear relationships between input and output data sets [51]. ANNs have been applied to multispectral information obtained from multiple types of sensors and platforms, for example, for multispectral imagery classification and segmentation [52,53]. Several ANN techniques were preferred over different spectral information capable of predicting firmness and soluble content in apple fruits [54], leaf recognition [55], crop nitrogen stress [56] and vegetation mapping [57]. Moreover, different types of plant stress have been detected using ANN and multispectral information [56,58,59,60]. Specifically, water stress has been assessed modelling thermal information using ANN [61] showing correlations between 0.89–0.93 in different cultivars.

Considering that conventional spectral indices do not accurately predict the spatial variability of Ψ_stem_, this study aimed to develop and validate ANN models to improve the prediction of the intra-vineyard spatial variability of Ψ_stem_ using multispectral information between 500–800 nm obtained from an UAV. As a reference, relationships between different conventional spectral indices and Ψ_stem_ were evaluated.

## 2. Materials and Methods

### 2.1. Site Description, Experimental Design and Plant Water Status Measurements

The field experiment was conducted in Talca, Maule Region, Chile (35°27′38″ LS 71°29′53″ LW) on Carménère vines grafted on Paulsen-1103. The vines were planted in 2007 in North–South oriented rows at 2.5 m × 1.5 m and trained on a vertical shoot positioned (VSP) system. The field location has a Mediterranean, semi-arid climate with a temperature average of 17.1 °C and annual rainfall of 679 mm. Field collection and flights were carried out in summer, which is predominantly dry and hot (2.2% of annual rainfall). Vineyard soil was classified as Talca series (fine, mixed, thermic Ultic Haploxeralfs) with a clay loam texture and an average bulk density of 1.5 g·cm^−3^. At the effective rooting depth (0–60 cm), the volumetric soil water content at field capacity and wilting point were 0.36 m^3^·m^−3^ and 0.22 m^3^·m^−3^, respectively. The vines were irrigated daily using 4 L·h^−1^ drippers spaced at intervals of 1 m.

**Figure 1:**
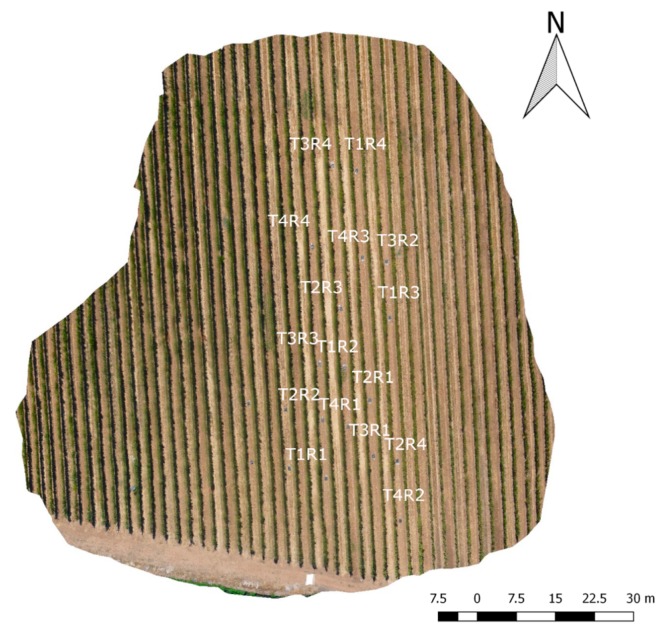
Treatments (T) and Repetitions (R) field distribution.

The experimental design was completely randomized with four different treatments with four repetitions (see Figure 1) and six plants per repetition. These treatments consisted of four stem water potential thresholds including non-water stress (T_0_) (Ψ_stem_ > −0.8 MPa), moderate water stress (T_1_) (Ψ_stem_ between −0.9 and −1.1 MPa), strong water stress (T_2_) (Ψ_stem_ between −1.2 and −1.4 MPa), and severe water stress (T_3_) (Ψ_stem_ < −1.4 MPa) [27]. A progressive water stress for each treatment was applied by stopping irrigation, and once the specific thresholds were reached, the irrigation was reestablished [62].

Ψ_stem_ was measured using a pressure chamber (PMS 600, PMS Instrument Company, Corvallis, OR, USA) from the middle trees of each repetition. A total of 32 leaves were measured corresponding to two mature and healthy sun-exposed leaves, sampled from the middle zone of the canopy which were covered with plastic bags and coated with aluminum foil for at least 1 h before measurements [22]. Ψ_stem_ was measured between 12:00 h and 14:00 h [63].

### 2.2. UAV Multispectral Image Acquisition

Five flights at an altitude of 60 m high were carried out during two seasons (three flights in 2014 and two in 2015) with the aim of extending the variability of field and plant condition. Meteorological conditions and phenological stages for each day of data collection were recorded (Table 2). All flights and image acquisition were concurrently done with Ψ_stem_ field measurements.

Flights in both seasons were carried out between 12:30 and 13:00, to reduce the ‘shadow-effect’ on the images [64]. Multispectral images were obtained from a MCA-6 camera (Tetracam’s miniature camera array), recording wavelengths at 530, 550, 570, 670, 700 and 800 nm. The image reflectance was normalized using a ‘white reference’ Spectralon panel (Labsphere Inc., Sutton, NH, USA) and compared with a spectroradiometer (SVC HR-1024, Spectra Vista Cooperation, Poughkeepsie, NY, USA) to account for any relative spectral response of each band of the camera as proposed by Laliberte, et al. [65]. All image processing was carried out using Matlab (MATLAB 2013a, The MathWorks, INC., Natick, MA, USA). The MCA-6 sensor was mounted on an octocopter, Mikrokopter Okto XL, equipped with the FlightNav 2.1 flight and navigation controller, MK3638 motors and 12”× 3.8’’ propellers. The sensor was affixed to a servo-controlled gimbal for stability and to ensure that it pointed directly down during flight.

### 2.3. Soil–Canopy Pixel Distinction

To separate canopy pixels a double normal distribution based on NDVI (normalized difference vegetation index) was built, because this index is related with vegetation structure [66]. From this distribution, one peak corresponded to soil and the other to canopy. The lower percentage of occurrence between both peaks which corresponded to neither canopy nor soil information was calculated. This NDVI value was used to apply a binary mask to the images with the aim of isolating the canopy and to extract pure plant spectral information.

Once the images were obtained and preprocessed, different spectral indices were calculated per pixel (6 × 6 cm^2^). Each experimental plot was isolated from the image, eliminating the border and separating soil from pure canopy information. This information was then correlated with field measurements of Ψ_stem_ to identify the indices that better represented the stem water potential. ANN models were tested to identify the best band combinations to simulate Ψ_stem_.

### 2.4. Artificial Neural Network (ANN) Computing

The first model was built including all bands (530, 550, 570, 670, 700 and 800 nm). Each band was then isolated to identify the best relationship between ANN and Ψ_stem_. A MultiLayer Perceptron (MLP) ANN type was used and a back-propagation process was carried out for weight calculations, in the same manner implemented in related studies [67,68,69].

To select the best combination of hidden nodes and number of iterations, we implemented the methodology developed by Ballesteros, et al. [70], which also avoided the problem of local convergence of the model. This methodology is based on evaluating the root mean square error (RMSE) with a trial-and-error method that implements the neural network with a wide range of hidden nodes (from two to twenty in one-node increments) and iterations (100–5000 in steps of 100 iterations). For each combination, the ANN was trained 20 times, which avoided the problem of local convergence. This method, although is computationally expensive, permits the clear detection of problems of local convergence that could appear when applying a back-propagation algorithm and overfitting the model [67,71].

### 2.5. Statistical Analysis

The data set was divided into calibration and validation, where 80% was used for the correlations and the ANN model for the calibration process, while the other 20% was used to validate the models. The validation set was obtained by random selection over a repetition of every treatment. The coefficient of determination (R^2^) was calculated to determine the linear correlation of Ψ_stem_ with the conventional indices. For the model validation, comparisons between observed and estimated values of Ψ_stem_ were carried out using the mean absolute error (MAE), root mean square error (RMSE), relative error (RE) and a modified index of agreement (d) [72].

## 3. Results

### 3.1. Soil–Canopy Pixel Distinction

**Figure 2:**
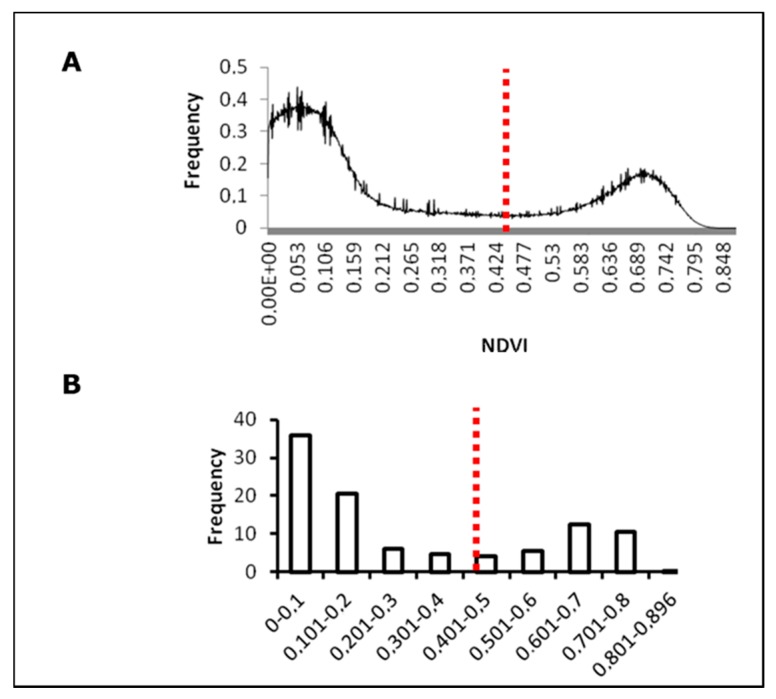
NDVI (Normalized Difference Vegetation Index) values distribution for soil-canopy pixels distinction. (**A**) NDVI frequency graph; (**B**) NDVI-ranged frequencies graph. Red line shows the NDVI threshold to separate canopy from soil.

**Figure 3:**
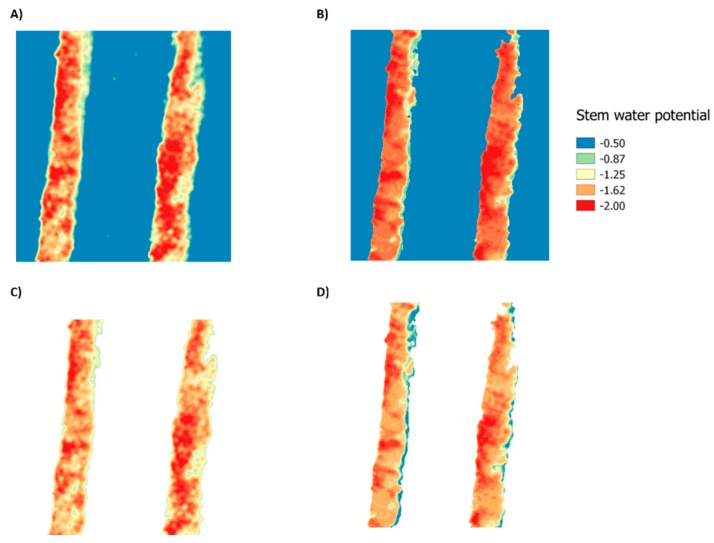
Examples of soil–canopy pixel distinction by the mask application based on the NDVI threshold for NDVI and artificial neural network model ANN-2. (**A**) NDVI soil–canopy information; (**B**) NDVI pure canopy; (**C**) ANN-2 soil–canopy information; (**D**) ANN-2 pure canopy.

The threshold value of NDVI to separate pure vegetation over other information was 0.46 (red line) with 0.029% of occurrence (Figure 2A) which corresponded to the lowest frequency of NDVI occurrence values with 0.45 (Figure 2B). To validate these results, fractional cover (fc) was calculated and compared with the methodology proposed by Ballesteros, et al. [73] who separate vegetation from soil using an ANN. Using the same set of images, the mean values of fc estimated using the methodology suggested by Ballesteros, et al. [73] and proposed in this study were 28.12 (± 0.4) and 28.32 (± 0.6)%, respectively. The NDVI threshold of 0.46 was selected to build a mask that was applied to the multispectral indices and ANN images. Figure 3 represents an example of pure canopy information for the NDVI and ANN model, based on the built mask.

### 3.2. Statistical Analysis for ANN Models and Spectral Indices

Statistical parameters for linear correlations between multispectral indices and midday stem water potential (Ψ_stem_) are presented in Table 3. There were significant linear correlations between Ψ_stem_ versus the conventional spectral indices NDVI (Normalized Difference Vegetation Index), GNDVI (Green Normalized Difference Vegetation Index) and MSR (Modified Simple Ratio) with values of R^2^ ranging between 0.31–0.35. For other multispectral indices, the regression analysis indicated that values of R^2^ were lower than 0.1.

The values of R^2^ for the ANN training process were between 0.56–0.87 (Table 4) with the best performance observed for the ANN-2 model, which included the bands 550, 570, 670, 700 and 800 nm.

Only the indices that were statistically representative (NDVI, GNDVI and MSR) were used in the model validation. The values of the index of agreement ranged between 0.51–0.54 while those of MAE, RMSE and RE were between 0.26–0.27 MPa, 0.32–0.34 MPa and −24.2–25.6%, respectively. The model validation indicated that ANN models were more accurate than the conventional indices, with indices of agreement ranging between 0.66–0.82. In this case, the ANN-2 model overestimated the values of Ψ_stem_ with values of MAE, RMSE, RE equal to 0.1 MPa, 0.12 MPa and −9.107%, respectively (Table 5).

**Figure 4:**
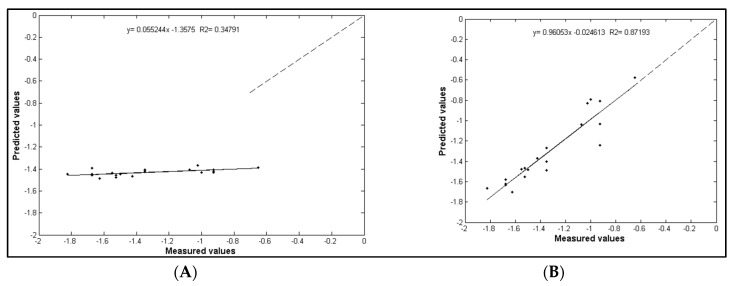
Comparison between estimated and measured values of midday stem water potential (MPa). (**A**) Normalized difference vegetation index (NDVI); (**B**) ANN-2 model.

When the estimated and observed values Ψ_stem_ for the ANN-2 and NDVI models were compared, the ANN-2 model was more accurate with a R^2^ = 0.87 and R^2^ = 0.35 respectively and closer to the 1:1 line (Figure 4).

**Figure 5:**
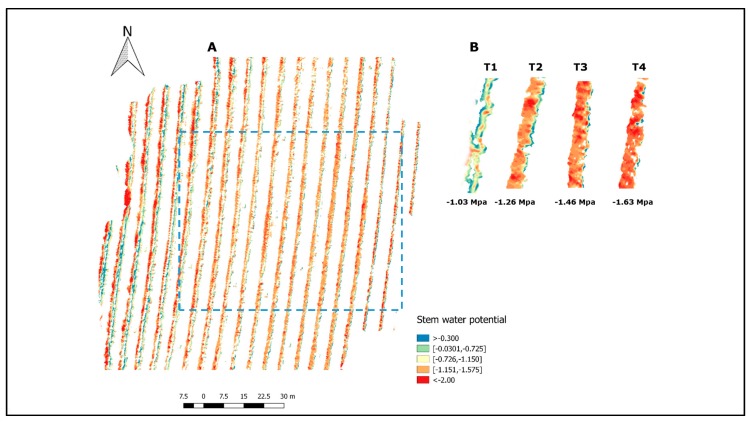
Predicted values of stem water potential (Ψ_stem_) for a whole flight. (**A**) All pure-canopy pixel information within the vineyard; (**B**) Predicted Ψ_stem_ classification within the four treatments.

**Figure 6:**
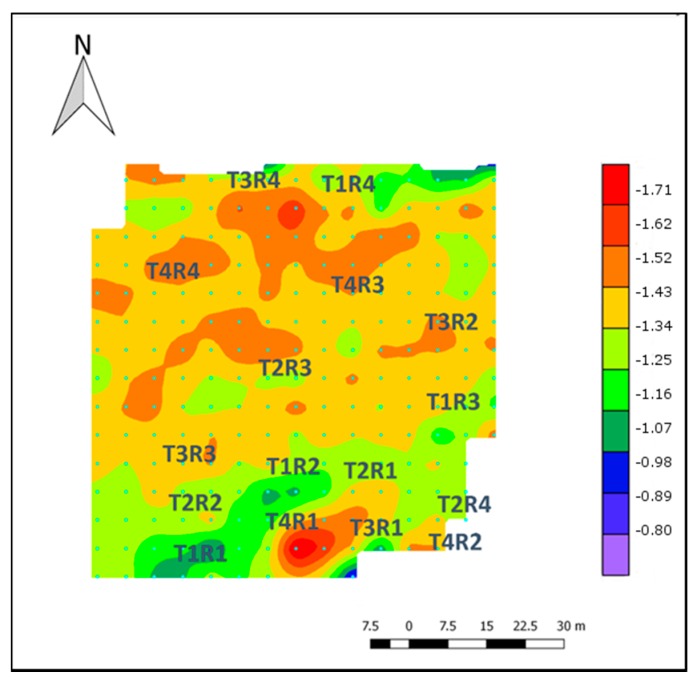
Intra-vineyard spatial variability of predicted midday stem water potential (Ψ_stem_) using an artificial neural network (ANN) model.

The ANN-2 model that included R550, R570, R670, R700 and R800 was applied to a whole flight and stem water potential was calculated applying the soil–plant filter described in the methodology. Figure 5A shows the prediction of Ψ_stem_ for each canopy pixel that was isolated by the soil–canopy distinction method used in this study. It represents the variability in the field considering a variation as small as 6 × 6 cm^2^. Based on Figure 5A,B, the differences caused by the water status treatments can be identified showing values that ranged between −0.3 and −2 MPa. Figure 5B represents the integration and classification of the information from the individual pixels for the whole treatment and the high contrast between non-stressed and severely-stressed plants can be analyzed. Although middle stress treatments do not show higher differences among them, they represent the transition between extreme treatments. Figure 6 represents the spatial variability of the field where the zones of different stress levels can be easily identified. The zones with higher levels of stress (T_3_ and T_2_ treatments) are represented in red and strong orange, while the treatments with lower levels of stress (T_0_ and T_1_) are represented in green. Based on Figure 6, moderate water stress can be identified for the majority of vines that were not part of the model’s construction.

## 4. Discussion

The results of conventional indices were consistent with the study carried out by Baluja, et al. [28], who indicated that higher correlations were observed for NDVI, GNDVI, TCARI/OSAVI and MSR with R^2^ ranging between 0.58 and 0.68. In this study, lower statistical values for correlation and validation between spectral indices and Ψ_stem_ were found, despite the inclusion of NIR and RED wavelength information, which have high reflectance on plant tissue [74] and a high absorbance by Chl [75], respectively. These indices can only indirectly detect water status differences, because they were developed to represent different physiological variables that can change according to different levels of water status. In this context, NDVI has been reported to be a good indicator of ‘vegetative expression’ [27] while GNDVI has been reported as a better form to detect Chl pigment concentration [76], which is modified under stress conditions. MSR was developed to improve the relationship of other indices with biophysical parameters in boreal forests [77]. TCARI/OSAVI was developed to make accurate predictions of crop Chl [78]. Furthermore, indices that use wavelength information between 500 and 800 nm have been reported by several studies with a high variation of R^2^ among vine cultivars when predicting water status. For example, TCARI/OSAVI showed the best correlation for 0.58 in Tempranillo [28], which decreased to 0.01 in Thompson Seedless [38]. NDVI showed the highest variation, reaching the lowest value of R^2^ = 0.03 in Cabernet Sauvignon [37]. This variation could be associated with the non-linear effect between water stress on different wavelength reflectances. The relationship between spectral indices and Ψ_stem_ is due to indirect changes produced by different levels of water stress, in contrast to thermal information, where direct effects such as stomatal closure can be assessed by thermal changes [64,68,79].

In this context, ANNs identify complex nonlinear relationships between input and output data sets [51] through input, hidden and output node layers [69]. That is the reason why ANNs have been used in several agricultural studies to analyze complex and non-linear relationships, such as ETo forecasting [80], rainfall–runoff modelling [51], rainfall forecasting [81], fruit firmness prediction [82], nitrogen stress identification [56], leaf recognition [55] and prediction of firmness and soluble content by using multispectral information [54]. ANN models have been proposed to better predict output variables compared with partial least-square (PLS) models [83], especially when NIR information is used [84,85].

Despite the advantages of ANN, several disadvantages and limitations are presented for these models. For example, when compared with linear relationships, building nonlinear models is inherently more difficult than linear ones [86]. Also, when machine learning models are applied to complex and high-dimensionality models, some criteria need to be accounted for. For example, the optimum dimensional reduction of classifiers is needed to improve classification [87,88,89]. Furthermore, Wu, et al. [81] and Taormina, et al. [90] suggested that inputs, modelling and data processing can be strongly improved if ANN models are coupled with preprocessing techniques [91]. Tu [92], made a comparison between logistic regressions and ANNs suggesting some disadvantage, such as (i) that the ‘black-box’ nature of ANNs has limited ability to explicitly identify possible causal relationships and cannot easily determine which variables are the most important contributors to a particular output; (ii) ANN development is a computationally intensive procedure that requires greater calculation time, which makes the portability application difficult when applying to the field; and (iii) due to the model interaction and nonlinearity, ANNs may cause overfitting of the training data set and produce poor performance in external sets (which are site-specific). However, Tu [92] suggested that this can be prevented by limiting the number of hidden nodes, adding a penalty term to the objective function for large weights and limiting the amount of training by cross-validation. In this case, dividing the dataset into calibration and validation data avoids overfitting problems. Zhang, et al. [93] suggested that ANN models are stronger than linear models when non-linear problems are studied and give several recommendations and examples of ANN model applications.

For the ANN-2 model, the exclusion of R530 is consistent with Rapaport, et al. [37], who found that at 530 nm the slope in relation to water status started to increase and reached the best correlation with Ψ_leaf_ at 538 nm. It has been proposed that at 531 nm nutrient and water stress can be detected, related to the xanthophyll cycle [94] due to a decrease in reflectance associated with a photoprotective response [95,96]. Despite this response, these changes can not always be identified in severely water stressed conditions [95,97]. In this context, Rapaport, et al [37] indicated that the information of 550, 570, 700 nm could better simulate different plant water statuses, which was included in the ANN-2 model. They also suggested that the NIR and RED wavelengths increased in all plant water statuses (control, light–moderate and severe stress). This can be understood as being due to the fact that 670 nm (RED) and 800 nm (NIR) wavelengths can detect plant tissue and have low and high reflectance in bushy (healthy) plants, respectively [98,99]. Since our study was carried out between two different seasons and in different months, we suggest that these wavelengths are relevant to representing all plant physiology differences presented in the field caused by the treatments. The final prediction for the whole flight (Figure 5 and Figure 6) represents the ability of the ANN-2 model to identify different contrasts of stem water potential present in the field. Also, the spatial variability of the Ψ_stem_ can be assessed and it is consistent with the field experimental design.

## 5. Conclusions

The utilization of UAV in obtaining high-resolution multispectral images and the use of artificial neural networks improved the assessment of the midday stem water potential spatial variability in a Mediterranean Carménère vineyard located in Talca, Maule Region, Chile. Artificial neural network models using information between 550–800 nm improved the Ψ_stem_ prediction showing values of R^2^, MAE, RMSE, RE equal to 0.87, 0.1 MPa, 0.12 MPa and −9.107%, respectively. As a future prospective, this study should be applied to a larger number of cultivars and fitted to different species to predict the spatial variability of water stress. Moreover, ANN-coupled models and different machine learning techniques should be implemented to assess improvements in the prediction of stem water potential spatial variability.

## Figures and Tables

**Table 1:** Conventional spectral indices used to estimate vine water status of different cultivars of *vitis vinifera*.

Index	Formula	R^2^	Reference	Cultivars
GI	$\frac{R_{550}}{R_{670}}$	0.54	[28]	*Vitis vinífera* L. cv tempranillo
GNDVI	$\frac{R_{800}-R_{550}}{R_{800}+R_{550}}$	0.58	[28]	*Vitis vinífera* L. cv tempranillo
MCARI	$\left[\left(R_{700}-R_{670}\right)-0.2\times \left(R_{700}-R_{550}\right)\right]\times \left(\frac{R_{700}}{R_{670}}\right)$	0.01	[28]	*Vitis vinífera* L. cv tempranillo
MCARI1	$1.2\times \left[2.5\times \left(R_{800}-R_{670}\right)-1.3\times \left(R_{800}-R_{550}\right)\right]$	0.21	[28]	*Vitis vinífera* L. cv tempranillo
MCARI2	$\frac{1.2\times \left[2.5\times \left(R_{800}-R_{670}\right)-1.3\times \left(R_{800}-R_{550}\right)\right]}{\sqrt{{\left(2\times R_{800}+1\right)}^{2}}-6\times \left(R_{800}-5\times R_{670}\right)-0.5}$	<0.01	[28]	*Vitis vinífera* L. cv tempranillo
MSAVI	$\frac{\left(2\times R_{800}+1-\sqrt{{\left(2\times R_{800}+1\right)}^{2}-8\times \left(R_{800}-R_{670}\right)}\right)}{2}$	0.11	[28]	*Vitis vinífera* L. cv tempranillo
MSR	$\frac{\left(\frac{R_{800}}{R_{670}}\right)-1}{\sqrt{\left(\frac{R_{800}}{R_{670}}\right)+1}}$	0.66	[28]	*Vitis vinífera* L. cv tempranillo
MTVI3	$1.2\times \left[1.2\times \left(R_{800}-R_{550}\right)-2.5\times \left(R_{670}-R_{550}\right)\right]$	0.01	[28]	*Vitis vinífera* L. cv tempranillo
NDVI	$\frac{R_{800}-R_{670}}{R_{800}+R_{670}}$	0.68 0.57 0.03	[28] [36] [37]	*Vitis vinífera* L. cv tempranillo *Vitis vinífera* L. cv chardonnay *Vitis vinífera* L. cv cabernet sauvignon
TCARI/OSAVI	$\frac{3\times \left[\left(R_{700}-R_{670}\right)-0.2\times \left(R_{700}-R_{550}\right)\times \left(\frac{R_{700}}{R_{670}}\right)\right]}{\left(1+0.16\right)\times \left(R_{800}-R_{670}\right)/\left(R_{800}+R_{670}+0.16\right)}$	0.58 0.01	[28] [38]	*Vitis vinífera* L. cv tempranillo *Vitis vinífera* L. cv thomson seedless
SRI	$\frac{R_{800}}{R_{550}}$	0.64	[28]	*Vitis vinífera* L. cv tempranillo
PRI	$\frac{R_{530}-R_{550}}{R_{530}+R_{550}}$	0.25 0.53 0.19	[28] [38] [37]	*Vitis vinífera* L. cv tempranillo *Vitis vinífera* L. cv thomson seedless *Vitis vinífera* L. cv cabernet sauvignon
RDVI	$\frac{R_{800}-R_{670}}{\sqrt{R_{800}+R_{670}}}$	0.10	[28]	*Vitis vinífera* L. cv tempranillo

GI = Green Index, GNVDI = Green Normalized Difference Vegetation Index, MCARI = Modified Chlorophyll Absorption in Reflectance Index, MSAVI = Improved SAVI Index, MSR = Modified Simple Ratio, MTVI3 = Modified Triangular Vegetation Index, NDVI = Normalized Difference Vegetation Index, TCARI/OSAVI = Transformed Chlorophyll Absorption in Reflectance index/Optimized Soil-adjusted Vegetation Index, SRI = Simple Ratio Index, PRI = Photochemical Reflectance Index, RDVI = Renormalized Difference VI.

**Table 2:** Air temperature (Ta), relative humidity (RH), wind speed (u) and phenological stage (PS) at the time of unmanned aerial vehicle (UAV) overpass during the 2014–2015 growing season.

Date	Flight Time (hh:mm)	Ta (°C)	RH (%)	u (Km/h)	PS
04/03/2014	13:00	21.3	52.5	5	Ripening
13/03/2014	12:30	21.6	54.3	3.5	Ripening
19/03/2014	12:45	21.3	51.4	3.5	Berry development
14/01/2015	12:30	25.2	49.7	6.8	Berry development
27/01/2015	12:30	24.4	41.2	7.4	Berry development

**Table 3:** Linear correlations between multispectral indices and midday stem water potential (Ψ_stem_).

Index	a	b	R^2^
NDVI *	−4.70	6.19	0.35
GNDVI *	−203.36	−140.75	0.31
PRI	−1.32	1.44	0.09
TCARI-OSAVI	−0.92	−0.74	0.09
GI	−2.03	1.40	0.06
MCARI	−1.27	−0.60	0.02
MCARI1	−1.22	−0.33	0.03
MCARI2	−1.43	0.03	<0.01
MSAVI	−1.31	−0.28	0.00
MSR *	10.78	8.45	0.34
MTVI3	−1.22	−0.33	0.03
SRI	−2.01	0.23	0.06
RDVI	−1.28	−0.35	0.00

* *p* < 0.05, a = intercept, b = slope.

**Table 4:** Values of coefficient of determination (R^2^) for the artificial neural network (ANN) model training.

ANN Model	Bands	R^2^
ANN-1 **	R_530_, R_550_, R_570_, R_670_, R_700_, R_800_	0.87
ANN-2 **	R_550_, R_570_, R_670_, R_700_, R_800_	0.87
ANN-3 **	R_530_, R_570_, R_670_, R_700_, R_800_	0.84
ANN-4 **	R_530_, R_550_, R_670_, R_700_, R_800_	0.78
ANN-5 **	R_530_, R_550_, R_570_, R_700_, R_800_	0.78
ANN-6 **	R_530_, R_550_, R_570_, R_670_, R_800_	0.68
ANN-7 **	R_530_, R_550_, R_570_, R_670_, R_700_	0.56

** *p* < 0.01.

**Table 5:** Statistical parameters of validation for conventional indices and artificial neural network (ANN) models.

Multispectral Index/ANNModel	MAE (MPa)	RMSE (MPa)	RE (%)	d
Multispectral indices				
NDVI *	0.25	0.32	−24.22	0.54
GNDVI *	0.27	0.34	−25.58	0.51
MSR *	0.26	0.33	−24.57	0.53
ANN models				
ANN-1 **	0.1	0.12	−9.21	0.82
ANN-2 **	0.1	0.12	−9.11	0.82
ANN-3 **	0.11	0.13	−9.68	0.8
ANN-4 **	0.12	0.15	−11.55	0.78
ANN-5 **	0.13	0.15	−11.61	0.77
ANN-6 **	0.15	0.2	−15.2	0.73
ANN-7 **	0.19	0.22	−16.5	0.66

MAE = mean absolute error, RMSE = root mean square error, RE = relative error, d = index of agreement. * *p* < 0.05, ** *p* < 0.01.
